# Prevalence of *Ureaplasma urealyticum*, *Chlamydia trachomatis*, *Neisseria gonorrhoeae* and herpes simplex virus in Beijing, China

**DOI:** 10.1017/S0950268818003163

**Published:** 2018-12-03

**Authors:** Y. Y. Liang, H. Y. Zhai, Z. J. Li, X. Jin, Y. Chen, S. P. Chen

**Affiliations:** 1 Department of Infection Control, Affiliated Hospital of Academy of Military Medical Sciences, Beijing, China; 2 Department of Laboratory Medicine, 307 Medical College of Anhui Medical University, Beijing, China; 3 Department of Laboratory Medicine, Affiliated Hospital of Academy of Military Medical Sciences, Beijing, China

**Keywords:** *Chlamydia trachomatis*, epidemiology, herpes simplex virus, *Neisseria gonorrhoeae*, *Ureaplasma urealyticum*

## Abstract

The prevalence of sexually transmitted infection (STI) pathogens in Beijing, China, is rarely reported. In this study, 34 911 symptomatic outpatients with suspected genital infections who attended outpatient clinics in a tertiary care hospital were included to investigate the updated prevalence of *Ureaplasma urealyticum* (UU), *Chlamydia trachomatis* (CT), *Neisseria gonorrhoeae* (NG) and herpes simplex virus (HSV) from 1 January 2013 to 31 December 2016 in Beijing, China. Results indicated that a decrease trend (UU, CT, NG and HSV) in male and an increase trend (UU, CT and NG) in female were observed during the period. Patients aged 20–39 years old were mostly affected by these pathogens, while the prevalence in patients aged 20–29 years old was the highest, The prevalence of UU in male was significantly lower than in female (31.5% *vs.* 49.3%, *P* < 0.05), while the prevalence of NG in male was significantly higher than in female (2.5% *vs.* 0.8%, *P* < 0.05). In patients with co-infections, 60.6% of male and 71.4% of female were co-infected by UU + CT. In total, 11.9% and 88.1% of patients with HSV infections were confirmed to be infected by HSV-1 and HSV-2. This study could contribute to a better understanding of the current epidemiological features of UU, NG, CT and HSV among symptomatic patients attending an outpatient clinic in Beijing, China, and thus facilitate to develop more effective intervention, prevention and treatment of STI.

## Introduction

Sexually transmitted infections (STIs), also referred to as sexually transmitted diseases or venereal diseases, are infections caused by pathogens which are commonly transmitted by sex, especially vaginal intercourse, anal sex or oral sex. According to the WHO data updated in August 2016, there are about 357 million new infections with chlamydia, gonorrhoea, syphilis or trichomoniasis, more than 500 million genital infections caused by herpes simplex virus (HSV), and more than 290 million women infections caused by human papillomavirus (http://www.who.int/mediacentre/factsheets/fs110/en/). Although some infections are curable (such as chlamydia, gonorrhoea, syphilis and trichomoniasis), they can cause acute conditions such as cervicitis, urethritis and genital ulceration, even lead to severe complications and long-term sequelae including pelvic inflammatory disease, ectopic pregnancy, infertility, neonatal death, premature delivery, blindness and cervical carcinoma [1–4]. *Ureaplasma urealyticum* (UU) is also confirmed to be an aetiological pathogen in nongonococcal urethritis (NGU) [5]. In addition, these STIs have also been shown to increase the risk of HIV acquisition and transmission [6–8].

Based on the prevalence data in general population from 2005 to 2012, the estimated global prevalence of chlamydia, gonorrhoea, trichomoniasis and syphilis is 4.2%, 0.8%, 5.0% and 0.5% in female, and 2.7%, 0.6%, 0.6% and 0.48% in male, respectively [9]. Before and during the early period of founding of China in 1949, syphilis, gonorrhoea and other STIs were very common in the country, and the prevalence of syphilis among sex workers is 84% in Beijing. Although syphilis had been eliminated from China in 1964 by free screening and treatment, STI re-emerges with rapid economic and social changes over the past three decades and rapidly spreads throughout the country from the early 1980s [10]. In 2001, NGU surpasses gonorrhoea and ranks first in eight common STI pathogens in China [11]. *Neisseria gonorrhoeae* (NG) resistance rates to penicillin, tetracycline and ciprofloxacin are also high in China [12, 13]. However, the majority of STI surveillance was focused on high-risk populations like female sex workers and their clients, men who have sex with men (MSM), drug users and youth [14]. The prevalence of STI pathogens in Beijing, China, is rarely reported.

The aim of this study was to characterise the prevalence of four common STI pathogens (UU; *Chlamydia trachomatis*, CT; NG; HSV) according to our retrospective analysis of outpatients from a tertiary care hospital in Beijing, China.

## Patients and methods

### Study populations

This study comprised of 34 911 outpatients (23 860 male, median age 31; 11 051 female, median age 33) with suspected genital infections who attended the outpatient clinics of department of gynaecology and department of urology in our tertiary care hospital (1500 beds) from 1 January 2013 to 31 December 2016. All patients complained of one or more urethral syndromes like vaginitis, cervicitis, dysuria, urinary frequency and pelvic inflammatory disease. After laboratory tests, patients could obtain effective symptomatic treatments accordingly. Patients who were tested twice or more times during the treatment process were only calculated for once as following: negativity was considered if all tests were negative, while positivity was considered if one test was positive.

### Specimen collection and detection of UU, CT, NG and HSV

The genital swabs were collected from outpatients by strictly aseptic manipulation. Swabs were placed into 2 ml of sterile normal saline and the tubes were vortexed. CT real-time polymerase chain reaction (PCR) kit, NG real-time PCR kit, UU real-time PCR kit and HSV I&II real-time PCR kit (Liferiver Biotech) were used for detection as per the manufacture's instruction. After September 2015, HSV I&II typing real-time PCR kits (Liferiver Biotech) were used for detection and typing. One millilitre of each sample was centrifuged at 13 000 ***g*** for 5 min at room temperature. The supernatant was discarded and the pellet was washed twice with normal saline. After the second washing, the pellet was resuspended in 100 µl extraction buffer and heated at 100 °C for 10 min. After another centrifugation at 13 000 ***g*** for 5 min, the supernatants were collected and used as DNA extracts.

The reaction mixtures were prepared as described by the manufacturer's instructions. The amplifications were performed on a Roche LightCycler^®^ 480 system in the following conditions: 94 °C 2 min, 40 cycles of 93 °C 15 s and 60 °C 60 s.

### Statistical analysis

All statistical analysis was performed using the SPSS software package version 20.0. Overall prevalence was calculated by dividing the number of positive patients by the number of tested patients. The age-adjusted prevalence in male or female was calculated according to the updated Beijing census population data (2010). Age was stratified into seven categories: 10–19, 20–29, 30–39, 40–49, 50–59, 60–69 and 70–79. The confidence intervals (CI) were calculated at the 95% level. Frequencies were compared using Pearson's *χ*^2^ analysis. *P* values of less than 0.05 were considered statistically significant.

## Results

A total of 34 911 outpatients (23 860 male and 11 051 female) were included. The overall prevalence of UU, CT, NG and HSV was 35.5% (8481/23 860) (95% CI 34.9%–36.2%), 7.7% (1827/23 860) (95% CI 7.3%–8.0%), 2.7% (654/23 860) (95% CI 2.5%–3.0%) and 1.2% (277/23 860) (95% CI 1.0%–1.3%) in male, and 58.0% (6414/11 051) (95% CI 57.1%–59.0%), 5.9% (650/11 051) (95% CI 5.4%–6.3%), 0.6% (66/11 051) (95% CI 0.5%–0.7%) and 1.1% (124/11 051) (95% CI 0.9%–1.3%) in female, respectively. The prevalence of co-infections (two pathogens or more) in male and female was 5.0% (1195/23 860) (95% CI 4.7%–5.3%) and 6.3% (695/11 051) (95% CI 5.8%–6.7%), respectively. The prevalence of mono-infection with UU in male and female was 31.8% (7593/23 860) (95% CI 31.2%–32.4%) and 52.5% (5806/11 051) (95% CI 51.6%–53.5%), respectively.

### Prevalence from 2013 to 2016

**Fig. 1.**
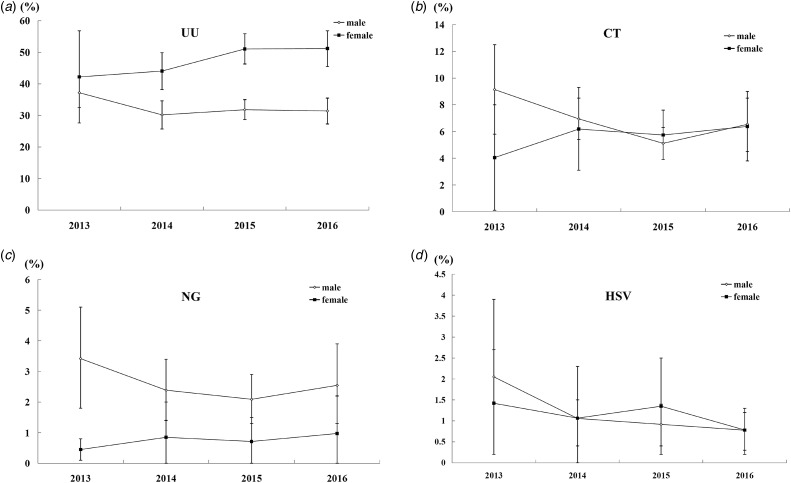
The adjusted prevalence of UU, CT, NG and HSV in male and female by calendar year.

In total, 3073, 7741, 8644 and 4402 male patients, as well as 629, 1933, 4501 and 3988 female patients, were tested from 2013 to 2016, respectively. As the adjusted prevalence showed in Figure 1, a decrease trend for the four pathogens (UU, CT, NG and HSV) in male and an increase trend for the three bacteria (UU, CT and NG) in female were observed. It was noteworthy that the prevalence of CT and NG in male increased in 2016 when compared with 2015 (*P* < 0.05).

### Age differences in prevalence

**Fig. 2.**
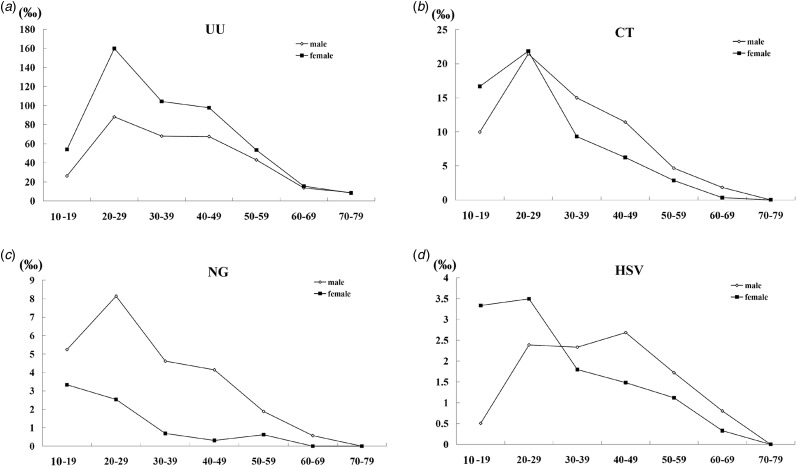
The adjusted prevalence of UU, CT, NG and HSV in male and female by age groups.

According to seven age categories of 10–19, 20–29, 30–39, 40–49, 50–59, 60–69 and 70–79, there were 485, 9970, 7612, 3654, 1549, 523 and 67 male patients, as well as 117, 3723, 3821, 2077, 1069, 209 and 35 female patients, were included in this study, respectively. The majority of patients infected by UU, CT, NG and HSV were those aged 20–39 years old, which was characterised by 73.5% (6234/8481) (95% CI 72.6%–74.4%), 79.5% (1452/1827) (95% CI 77.6%–81.3%), 77.5% (507/654) (95% CI 74.3%–80.7%) and 68.2% (189/277) (95% CI 62.7%–73.8%) for male, 71.7% (4598/6414) (95% CI 70.6%–72.8%), 80.2% (521/650) (95% CI 77.1%–83.2%), 78.8% (52/66) (95% CI 68.7%–88.9%) and 72.6% (90/124) (95% CI 64.6%–80.5%) for female, respectively. As shown in Figure 2, there was a trend that adjusted prevalence of UU, CT, NG or HSV increased to the highest for patients aged 20–29 years old, then decreased over time, regardless of gender. The exception was longer age span for HSV in male (from aged 20–29 to aged 40–49) and no increase for NG in female.

### Gender differences in prevalence

**Fig. 3.**
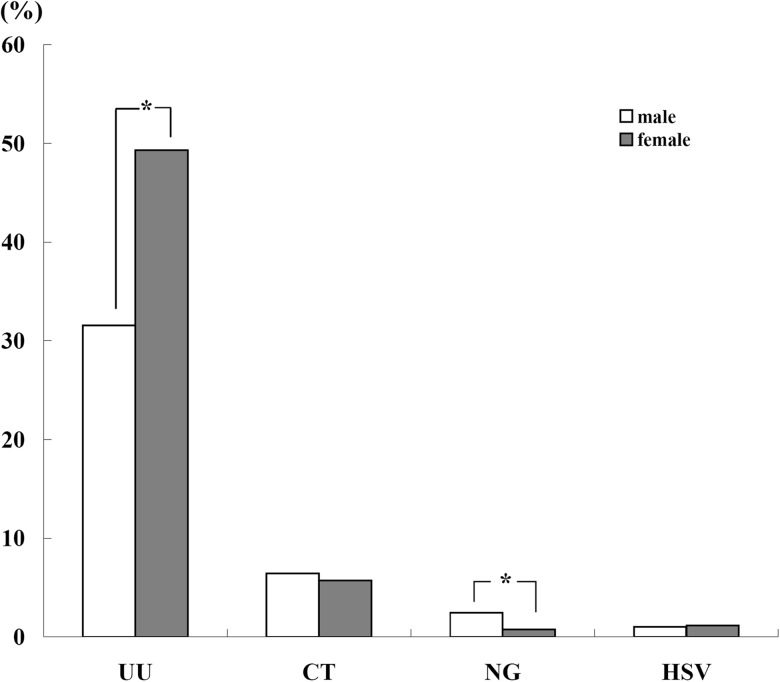
The adjusted overall prevalence of UU, CT, NG and HSV in male and female.

Among 23 860 male and 11 051 female patients, the adjusted prevalence of UU in male was significantly lower than that in female (31.5% *vs.* 49.3%, *P* < 0.05). Contrarily, the adjusted prevalence of NG in male was significantly higher than that of female (2.5% *vs.* 0.8%, *P* < 0.05) (Fig. 3). There was no significant difference in the adjusted prevalence of CT and HSV between male and female (*P* > 0.05) (Fig. 3).

### Prevalence of co-infections

**Fig. 4.**
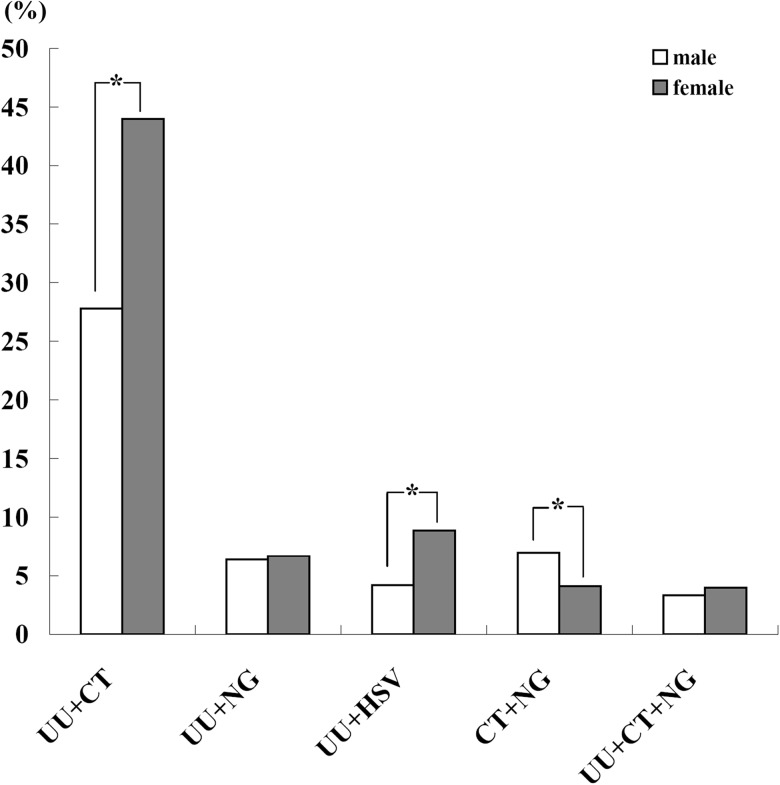
The adjusted overall prevalence of co-infections in male and female.

Only co-infections with UU + CT, UU + NG, UU + HSV, CT + NG and UU + CT + NG were identified in this study. In those patients with co-infections, 60.6% (724/1195) (95% CI 57.8%–63.4%) of male and 71.4% (496/695) (95% CI 68.0%–74.7%) of female were co-infected by UU + CT. The adjusted prevalence of UU + CT and UU + HSV in female was significantly higher than those in male (44.0% *vs.* 27.8%, 8.9% *vs.* 4.2%; *P* < 0.05), while the adjusted prevalence of CT + NG in female was significantly lower than those in male (4.1% *vs.* 7.0%, *P* < 0.05) (Fig. 4). There were no significantly differences in the adjusted prevalence of UU + NG and UU + CT + NG between male and female (*P* > 0.05) (Fig. 4).

### Association between HSV type and STI

From September 2015 to December 2016, the HSV I&II typing real-time PCR kit, instead of the universal HSV I&II real-time PCR kit, was used to distinguish infections caused by HSV-1 or HSV-2. In the 12 218 tested patients, HSV types were confirmed in 118 patients which was characterised by 11.9% (14/118) (95% CI 5.9%–17.8%) of HSV-1 and 88.1% (104/118) (95% CI 82.2%–94.1%) of HSV-2.

## Discussion

STIs remain major significant public health problems worldwide [9]. In China, STIs rapidly spread since China's reform and opening policy in the 1980s. Female sex workers and their clients, MSM, drug users, migrant workers and youth are considered as high-risk groups of STIs [14]. Thus, there is an urgent need to prevent the spread of STIs from these high-risk populations to the general populations in China. However, the epidemiology of STI pathogens in Beijing, China, is rarely described [10]. This study retrospectively investigates the epidemiology of UU, CT, NG and HSV in consecutive outpatients visited our hospital from 2013 to 2016, and thus will provide updated STI information for the government to take measures to control the spread of sexually transmitted diseases.

A National System of STIs Surveillance was initiated to monitor the magnitude of STIs and its trend in China in 1988. Based on data retrieved from the surveillance system, the epidemiology of STIs from 1989 to 1998 is described in a previous study which shows an increasing prevalence of gonorrhoea, syphilis, genital warts and NGU in patients [10]. However, the detailed epidemiological characteristics (such as differences in age, gender or calendar year) of each pathogen are unclear. Our study detailedly investigates the epidemiology of four common STI pathogens (UU, CT, NG and HSV) from 2013 to 2016 in Beijing, China. Compared with the previous study [10], the overall prevalence presented here shows a significant decrease in the prevalence of NG. It's also important to note that a decrease trend of prevalence for UU, CT, NG and HSV is observed in male. On the contrary, an increase trend of prevalence for UU, CT and NG is observed in female.

For both male and female, a strong risk factor associated with CT and NG infections is young age [15, 16]. Our results clearly showed that youth between 20 and 39 years old are largely affected by STI which is also consistent with a previous study performed in China [10]. However, the prevalence in youth between 20 and 29 years old is the highest regardless of gender or pathogen (except for NG in female). It is noteworthy that one male who is aged 14 years is co-infected by UU, CT and NG. The correlation between young age and STI might be due to biological and behavioural factors. First, younger individuals are sexually active. Second, younger individuals engage in more risky sexual behaviours like multiple sexual partners, MSM and unprotected sex. Thus, we strongly recommend urgent sex education programmes and STI screening for youth aged 20–29 years old to prevent the spread of STI pathogens, as well as to provide better cost savings due to early diagnosis and treatment.

Genital herpes is a common sexually transmitted disease caused by HSV-1 or HSV-2. Typically, HSV-1 causes non-sexually-transmitted oral herpes infections. However, oral sex can increase the possibility and incidence of primary genital infections by HSV-1 [17–19]. In this study, 11.9% (14/118) of HSV-positive patients were confirmed to be infected by HSV-1, which suggests that HSV-1 has become a common STI pathogen and that unprotected oral sex might have existed as one of socially accepted sexual behaviours in China [20, 21]. Effective control measures, such as male or female condoms, topical microbicides and partner notification, can be taken to prevent the transmission of HSV.

UU is confirmed to be an aetiological pathogen in NGU [5]. In this study, the prevalence of UU in female is significantly higher than male, which is consistent with previous studies [22–24]. Although UU is previously considered as a non-pathogenic bacterium in female [25], the inconsistent results might be caused by low UU loads in those female patients [26]. Higher UU prevalence in female might be due to their different structures of genital tracts and endocrine factors. The better environment (pH, temperature and humidity) of female genital tracts is more suitable for propagation of bacteria. However, the NG prevalence in male and female is contradictory up to now [27–31]. Our results show a NG infection bias towards male which are consistent with some previous studies [29–31], while some other studies indicate a higher prevalence in female [27, 28]. The discrepancies are not addressed, but participant characteristics (age, race, risk population, etc.), detection methods (based on DNA or RNA) and statistical analysis (models, data weighted or not) might bias the results.

Our study has three limitations. First, a weakness of our study is that it comes from a single hospital. Larger sample and multi-centres (located in different geographical areas) studies including more STI pathogens are needed to investigate the epidemiology more rigorously. Second, because pharyngeal and rectal swabs are not available in this study, the NG prevalence might be underestimated due to higher prevalence in the risk population like MSM and sex workers. Third, this is a retrospective study and all symptomatic individuals are outpatients from department of gynaecology and department of urology. No interviews or questionnaires are completed to obtain patients’ information like marriage, education, condom usage, sexual history, sexual partners, sexual debut, sexual orientation, STI history, and drug usage, which might bias the prevalence estimates presented here. In addition, the demographic information could be used to identify more risk factors for these STI pathogens in Chinese.

In conclusion, youth between 20 and 39 years old are mostly affected by the four common STI pathogens (UU, CT, NG and HSV), while the prevalence of those between 20 and 29 years old is the highest; NG infections are biased towards male and UU infections are biased towards female; HSV-1 has become a common STI pathogen in Beijing, China; as to the prevalence of UU, CT, NG and HSV from 2013 to 2016, there is a decrease trend and an increase trend in male and female, respectively (except for HSV in female). This study could contribute to a better understanding of the current epidemiological features of four common STI pathogens (UU, NG, CT and HSV) in Beijing, China, and thus facilitate to develop more effective intervention, prevention and treatment of STI.
